# In-Situ TEM Annealing Observation of Helium Bubble Evolution in Pre-Irradiated FeCoNiCrTi_0.2_ Alloys

**DOI:** 10.3390/ma14133727

**Published:** 2021-07-02

**Authors:** Huanhuan He, Zhiwei Lin, Shengming Jiang, Xiaotian Hu, Jian Zhang, Zijing Huang

**Affiliations:** College of Energy, Xiamen University, Xiamen 361005, China; 32420181153444@stu.xmu.edu.cn (H.H.); 32420181153424@stu.xmu.edu.cn (Z.L.); 32420191152363@stu.xmu.edu.cn (S.J.); 32420201152830@stu.xmu.edu.cn (X.H.)

**Keywords:** FeCoNiCrTi_0.2_, high-entropy alloys, He^+^ ions irradiation, helium bubbles

## Abstract

The FeCoNiCrTi_0.2_ high-entropy alloys fabricated by vacuum arc melting method, and the annealed pristine material, are face centered cubic structures with coherent γ’ precipitation. Samples were irradiated with 50 keV He^+^ ions to a fluence of 2 × 10^16^ ions/cm^2^ at 723 K, and an in situ annealing experiment was carried out to monitor the evolution of helium bubbles during heating to 823 and 923 K. The pristine structure of FeCoNiCrTi_0.2_ samples and the evolution of helium bubbles during in situ annealing were both characterized by transmission electron microscopy. The annealing temperature and annealing time affect the process of helium bubbles evolution and formation. Meanwhile, the grain boundaries act as sinks to accumulate helium bubbles. However, the precipitation phase seems have few effects on the helium bubble evolution, which may be due to the coherent interface and same structure of γ’ precipitation and matrix.

## 1. Introduction

Recently, high-entropy alloys (HEAs) have been regarded as one of the potential nuclear structural candidate materials due to their excellent irradiation resistance [1,2,3,4,5]. In nuclear reactors, (n, α) a transmutation reaction will produce a number of helium atoms, which are easy to precipitate at grain boundaries and dislocations, forming helium bubbles and voids, resulting in material swelling and material failure [6]. At present, some progress has been made in the diffusion of He atoms and the growth mechanism of helium bubbles in high-entropy alloy materials [7,8,9,10,11,12]. Meanwhile, FeCoNiCr has been proven by its excellent resistance to heavy ions and He-ion irradiation [8,13]. Duan et al. found that the ultra-fine grained FeNiCoCr alloys remains stable under the irradiation damage up to 58 dpa in the temperature of 573 K and 773 K, which indicated that an excellent irradiation resistance of Fe-Co-Ni-Cr HEAs [13]. Moreover, previous studies have shown that the addition of Ti can form γ’ precipitations in FeCoNiCr HEAs, which will enhance the strength of FeCoNiCr HEAs. Han et al. observed the composition evolution of the γ’ precipitations at different aging duration in FeCoNiCrTi_0.2_ alloys, and proved that the γ’ precipitations have a (CoFeCrNi)_3_Ti phase [14]. Chen et al. found that 1/3 <111> dislocation loops were formed and the helium bubbles were observed under the irradiation of 275 keV He^+^ at 673 K [15]. Additionally, they also compared the FeCoNiCrTi_0.2_ alloys with the FeCoNiCr alloys and FeCoNiCr(Al/Cu)_0.2_ HEAs under He^+^ ion irradiation [16]. However, the size of γ’ precipitates is below 10 nm in those studies, where the interface effects of precipitates and matrix is hard to distiguish in TEM samples with ~100 nm thickness. Meanwhile, the evolution of the helium bubbles is rarely studied by in situ annealing experiments. Therefore, this article studies the He^+^ ion irradiation on FeCoNiCrTi_0.2_ alloys with larger size γ’ precipitations and performs the post-irradiation in situ annealing observation in order to better understand the influence of the γ’ precipitations on the behaviors of the helium bubbles.

## 2. Materials and Methods

The HEA FeCoNiCrTi_0.2_ were fabricated by vacuum arc melting with a mixture of pure metals (purity > 99.99 wt.%). These ingots were remelted at least five times to homogenize the ingredients and were drop-cast into a copper mold. The ingots were subsequently annealed at 1423 K for 2 h in a purity 99.99% Ar atmosphere and cooled in the furnace. Before He^+^ irradiation, the annealed samples were ground to a thickness of about 30 μm, punched into disks of 3 mm diameter and then thinned by twin-jet electro-polishing with a voltage of 20~30 V in a solution of 5% perchloric acid + 35% N-butanol + 60% Ethanol at the temperature of about −40 °C.

The well-polished tansmission electron microscope (TEM) samples were irradiated with 50 keV He^+^ ions at 723 K to a total fluence of 2 × 10^16^ ions/cm^2^ with ion flux of ~1.85 × 10^12^ ions/(cm^2^·s) by the NEC-400 kV ion implanter at Xiamen University (National Electrostatics Corp., Middleton, WI, USA). In situ TEM annealing experiments were performed at a temperature of 823 K for 30 min, followed by heating to 923 K for 30 min at an increasing temperature rate of 50 K/min, using a Gatan 652 double-tilt heating holder. Characterizations of pristine samples and helium-bubble evolution are examined by an FEI Tecnai F30 microscope with a double-tilt specimen holder, operated at 300 kV, where the degree of under-focus is about 3 μm to observe the helium bubbles.

## 3. Results and Discussion

**Figure 1:**
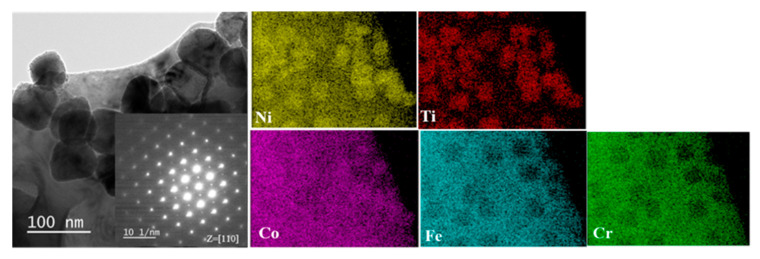
The micro-structure of pristine FeCoNiCrTi_0.2_ alloys under TEM characterizations.

Figure 1 presents the TEM microstructures of the annealed FeCoNiCrTi_0.2_ samples, including bright-field image and its corresponding selected area electron diffraction (SAED) pattern and EDS results from a region containing the matrix and precipitates. In the bright-field image, the precipitates of approximately 60–80 nm can be clearly observed. The corresponding SAED pattern along zone axis z = [110] shows that the precipitates have an L1_2_ structure (γ’ phase), which is completely coherent with the face centered cubic crystal structure of matrix with a lattice constant of 3.6061 Å. The STEM-EDS results indicated that the main elements of the γ’ precipitation are Ni and Ti; the element Co is partitioned into the γ’ precipitation, the distribution of elements Fe and Cr is poor. The atomic percentage of the Ni and Ti elements is about 3:1. These results are consistent with the previously reported structure of FeCoNiCrTi_0.2_ samples [14].

**Figure 2:**
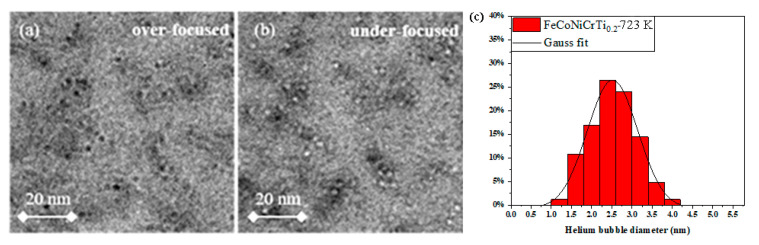
Distribution of helium bubbles and Gaussian fitting in the FeCoNiCrTi_0.2_ alloys after pre-irradiation at 723 K. (**a**) over-focused; (**b**) under-focused; (**c**) He bubbles distribution.

Figure 2 shows the helium bubbles and defects generated inside the FeCoNiCrTi_0.2_ samples under He^+^ ions irradiation at 723 K. To better examine the distribution and size of helium bubbles produced by He^+^ irradiation, the TEM images are performed under under-focus and over-focus, respectively. In Figure 2a, helium bubbles appear as black dots with white edges at the over-focus state. In contrast, helium bubbles are white dots with black edges under the under-focus condition, shown in Figure 2b. Inside the FeCoNiCrTi_0.2_ samples, the helium bubbles present as dispersed distribution and the helium bubbles near the defects tend to gather together. The diameter distribution of helium bubbless in Figure 2a,b can be fitting well with Gaussian distribution (see Figure 2c), and the mean diameter is about 2.5 ± 0.47 nm.

**Figure 3:**
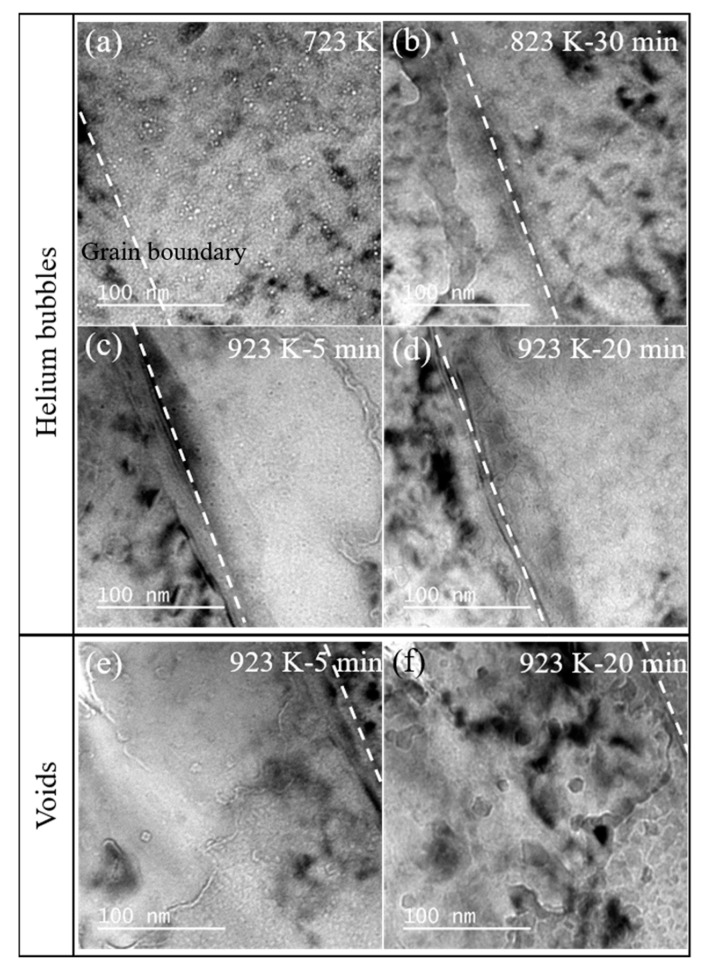
(**a**–**d**) The evolution of helium bubbles during in situ annealing at 823 K and 923 K; (**e**–**f**) the evolution of voids at 923 K in the FeCoNiCrTi_0.2_ alloys.

Figure 3 shows the evolution of helium bubbles observed at the grain boundary during in situ annealing at the 823 K and 923 K. Before annealing (Figure 3a), the helium bubbles near the defects are more concentrated, which is the same as the result in Figure 2. After annealing at 823 K for 30 min (Figure 3b), the number of helium bubbles decreases and the helium bubbles tend to move to the grain boundary labeled with white broken lines in Figure 3. The annealing time has an influence on the nucleation and growth of helium bubbles, and some of the helium bubbles diffuse and release to the free surface. Actually, the grain boundary and the sample surface can act as the effective sinks for helium atoms. After holding at 923 K for 5 min, there are more bubbles at the grain boundary than inside the grain (Figure 3c). After the annealing time reaches 20 min at 923 K, the surface is completely destroyed (Figure 3b). In the in situ annealing process, the size of the helium bubbles gradually increases with the increase in the holding time and heating temperature. The specific values of the helium bubbles size are summarized in Table 1. Meanwhile, the helium bubble density will decrease with the annealing time during the migration and coalescence process. The areal densities of helium bubbles are summarized in Table 1. It was found that the helium areal density will decrease slowly at 823 K with annealing time, while the helium areal density will substantially decrease with the annealing temperature increase to 923 K.

In addition, the distribution of helium bubbles in the observed irradiation area is relatively homogeneous, as shown in Figure 2 and Figure 3. This indicates that the 60–80 nm γ’ precipitations seem have few effects on the helium bubbles’ evolution, which may be due to the coherent interface and same structure of γ’ precipitation and matrix. On the other hand, the γ’ precipitations can enhance the strength of the alloys without affecting the evolution of the helium bubbles, which provides us with new prospects for high strength and radiation-tolerant materials design.

At 923 K, the generation and evolution of voids with quadrilateral or hexagonal shapes are observed during annealing, compared with annealing at 823 K where the evolution of helium bubbles is only observed at 823 K. The size and number of voids increase as the holding time increases, as shown in Figure 3e–f.

**Figure 4:**
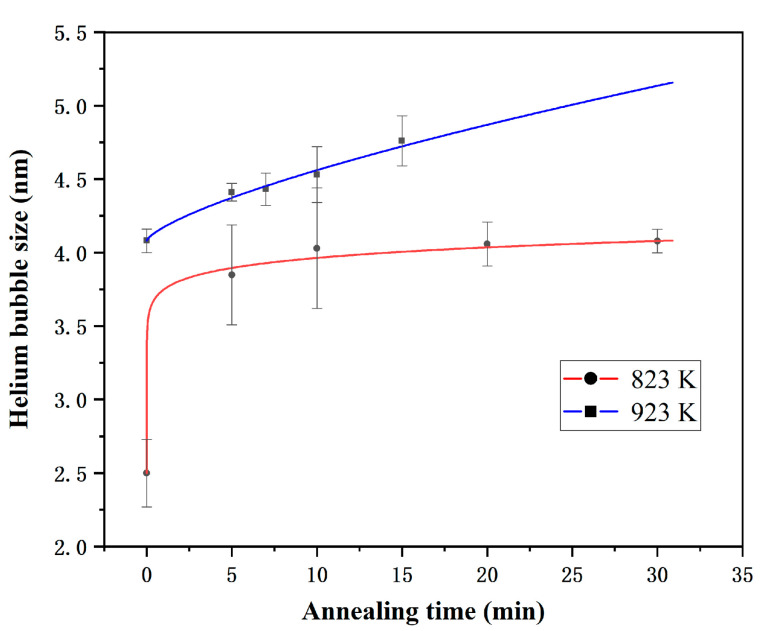
Evolution of He bubbles sizes with annealing times at different temperatures.

According to the above results, the helium bubble sizes at different temperatures are plotted and fitted with annealing time to further explore the helium bubble evolution behaviors in Figure 4. At given temperatures and helium concentrations, the relationship between the annealing time and the size of helium bubbles can be expressed by the following power function [17]:

(1) $$R_{b\;}{=A\;\times \;t}^{n}{+R}_{0}$$

where $R_{b}$ is the mean bubble diameter in nanometers at temperature b,t is the annealing temperature, and $R_{0}$ is the original bubble diameter. The final fitting formula is as follows:

(2) $$R_{823\;K}=1{.25388\;\times \;t}^{0.06887}+2.5$$

(3) $$R_{923\;K}=0{.09236\;\times \;t}^{0.71588}+4.08$$

Obviously, the longer the annealing time, and the higher the annealing temperature, the faster the growth rate of helium bubbles. Previous studies prove that the coarsening mechanism of helium bubbles is divided into two types: migration and coalescence (MC) and Ostwald ripening (OR) [18]. Additionally, the melting point of FeCoNiCr is around 1695 K [19], which means that the experimental temperature in this study is between 0.2–0.5 T_m_. In this study, the migration and coalescence (MC) mechanism is effective at the temperatures of 0.2–0.5 T_m_ [11]. The temperature dependence on the final diameter follows an Arrhenius relationship [20]:

(4) $${D=D}_{0}\;\exp\;(-\frac{E_{a}}{kT})$$

where D is the bubble diameter, k is the Boltzmann constant, $E_{a}$ is the effective activation energy of coarsening, and T is the annealing time. The effective activation energy $E_{a}$ of bubble coarsening can be calculated as 1.638 eV. Meanwhile, a multi-element nucleation model is proposed to describe the stability of helium bubbles, as follows [8,12]:

(5) $$C_{B}\left(t\to \infty \right){\sim 2C}_{B}^{*}\propto {\left(\frac{P_{He}}{t^{\gamma }c_{Hel}^{*}}\right)}^{\frac{1}{\gamma +1}}{/D}_{Hel}$$

(6) $$\bar{r_{B}}\left(t\to \infty \right)\propto {{[D}_{Hel}c_{Hel}^{*}{}^{1/(\gamma +1)}{\left(\frac{P_{\mathrm{He}}}{t^{\gamma }}\right)}^{\frac{\gamma }{\gamma +1}}t^{\gamma +1}]}^{1/\beta }$$

where $C_{B}$ is the density of helium bubbles, C_Hel_^*^ is the helium concentration in solution at nucleation peak, $\bar{r_{B}}$ is the mean diameter of helium bubbles, $P_{He}$ is the production rate of helium atoms, t is the helium bubble evolution time. The formula shows that the larger of the helium bubble diffusion coefficient ($D_{Hel}$), the larger of the helium bubble size and the lower of the bubble density. The increase in annealing temperature results in a larger helium bubble diffusion coefficient and a larger bubble size, which is consistent with our experimental results.

Previous analysis illustrated that the experimental temperature is approximately 0.2–0.5 T_m_, which means that migration rate of vacancies caused by irradiation and thermal effect will be significantly improved. Trinkaus et al. counted and proved that the diffusion of helium atoms is dominated by the vacancy mechanism at the temperature of 0.2–0.5 T_m_ [11]. After reaching this temperature, the vacancies in the sample quickly migrate and merge to form voids. Meanwhile, the size and number of cavities increase as the holding time increases. In addition, the FeCoNiCrTi_0.2_ samples possess an FCC structure containing γ’ precipitation with a coherent L1_2_-Ni_3_Ti structure with the matrix. Total dislocations with a Burgers vector a/2⟨110⟩ and partial dislocation including Shockley partial dislocation and Frank partial dislocation will be introduced by irradiation. In FCC-structure crystals, Shockley partial dislocation possess a burgers vector $a/6\langle 11\bar{2}\rangle$ and Frank partial dislocation is a/3⟨111⟩ on the {111} crystal plane. These dislocations in different directions react with each other to form a tetrahedron or octahedron, resulting in the quadrilateral or hexagonal voids shape.

## 4. Conclusions

In summary, we have investigated the process of bubble evolution and formation under 50 keV He^+^ irradiation at 723 K in FeCoNiCrTi_0.2_ alloys. Helium bubbles were observed when the samples were pre-irradiated at 723 K. The diameter of helium bubbles gradually increased and fit well with the Gaussian distribution. The 60–80 nm γ’ precipitations seem have few effects on the helium evolution, which may be due to the coherent interface and same structure of γ’ precipitation and matrix. Additionally, the helium bubble sizes at different temperatures are plotted and fit well with the empirical power formula. Meanwhile, the migration and coalescence (MC) mechanism is applied to interpret the in situ annealing observations in this study. In addition, the generation and evolution of voids were observed during annealing at 923 K.

## Figures and Tables

**Table 1:** The size of helium bubbles under different annealing times at 823 K and 923 K.

823 K	Annealing time (min)	5	10	20	30
	Helium bubble size (nm)	3.85 ± 0.34	4.03 ± 0.41	4.06 ± 0.15	4.08 ± 0.08
	Areal density of helium bubbles (nm^−2^)	1.9 × 10^−2^	2.2 × 10^−2^	1.43 × 10^−2^	1.01 × 10^−2^
923 K	Annealing time (min)	5	7	10	15
	Helium bubble size (nm)	4.41 ± 0.06	4.43 ± 0.11	4.53 ± 0.19	4.76 ± 0.17
	Areal density of helium bubbles (nm^−2^)	6.1 × 10^−3^	6.0 × 10^−3^	5.1 × 10^−3^	4.2 × 10^−3^

## Data Availability

The data presented in this study are available on request from the corresponding author.
